# The effects of data-driven learning activities on EFL learners’ writing development

**DOI:** 10.1186/s40064-016-2935-5

**Published:** 2016-08-04

**Authors:** Qinqin Luo

**Affiliations:** School of Foreign Languages, Southwest Petroleum University, Chengdu, 610500 China

**Keywords:** Data-driven learning, EFL learners, Writing development, Corpus

## Abstract

Data-driven learning has been proved as an effective approach in helping learners solve various writing problems such as correcting lexical or grammatical errors, improving the use of collocations and generating ideas in writing, etc. This article reports on an empirical study in which data-driven learning was accomplished with the assistance of the user-friendly BNCweb, and presents the evaluation of the outcome by comparing the effectiveness of BNCweb and a search engine Baidu which is most commonly used as reference resource by Chinese learners of English as a foreign language. The quantitative results about 48 Chinese college students revealed that the experimental group which used BNCweb performed significantly better in the post-test in terms of writing fluency and accuracy, as compared with the control group which used the search engine Baidu. However, no significant difference was found between the two groups in terms of writing complexity. The qualitative results about the interview revealed that learners generally showed a positive attitude toward the use of BNCweb but there were still some problems of using corpora in the writing process, thus the combined use of corpora and other types of reference resource was suggested as a possible way to counter the potential barriers for Chinese learners of English.

## Background

Data-driven learning (DDL), involving the direct or indirect application of corpus technology, has received considerable attention among researchers and teachers over the past two decades. Johns and King (1991), being generally regarded as the most influential advocates of DDL, defined DDL as “the use in the classroom of computer-generated concordances to get students to explore regularities of patterning in the target language, and the development of activities and exercises based on concordance output”. Later, DDL was interpreted in various ways by different researchers thus there seemed to be no single and watertight definition of DDL (Boulton 2011). However, it could be found that the common aspects of DDL are the use of authentic corpus data and student-centered exploratory learning activities (e.g. Boulton 2009, 2010, 2011; Smart 2014). Compared with the traditional rule-based language learning approach, DDL has its own advantages in the following aspects: Firstly, DDL is based on naturally-occurring language in corpora, which can provide authentic input for learners. Secondly, DDL promotes learners’ active involvement in the learning process, which usually requires learners to discover or explore language rules by themselves based on their observation and analysis of the concordance output. Thirdly, unlike the rule-based language learning tending to separate grammar and lexis, DDL fosters a more lexico-grammatical approach by allowing students to use a concordancer to retrieve frequently occurred lexical or grammatical patterns for a search item (Flowerdew 2015). Due to the above advantages, DDL is suggested as effective in promoting foreign language or second language (L2) learning. The earlier studies about DDL were mostly theoretical but recent years have witnessed an increasing number of empirical studies about DDL. These studies have generally confirmed the positive sides of DDL in various aspects of language learning, such as promoting learner autonomy, increasing language awareness, enhancing noticing skills, extending learners’ cognitive abilities, etc.

Writing is one of the aspects where DDL has made a significant impact. The benefits of DDL in foreign language/L2 writing have been proved by a large number of studies (Gaskell and Cobb 2004; Chambers and O’Sullivan 2004; O’Sullivan and Chambers 2006; Yoon 2008; Gilmore 2009; Smart 2014; Tono et al. 2014; Chang 2014). However, the limitations of DDL, such as time-consuming and too high requirements for learners, have been also reported. To maximize the positive learning outcomes of DDL activities, several variables should be considered while designing DDL activities. The present study firstly analyzes the important factors that may influence the learning outcomes by reviewing relevant literature, and then an empirical study, aiming to investigate the medium-term effects of DDL activities in writing, is reported and discussed.

## Literature review

Foreign/second language writing usually poses great challenges to English as a foreign language (EFL) learners, whose writing are usually regarded as “non-nativelike”. The writing problems, such as lexical poverty and miscollocations, are commonly seen in their writing. DDL, a corpus-assisted language learning approach, was considered as advantageous in solving learners’ writing problems. But to ensure the effectiveness of DDL activities, a careful design should be considered. An examination of previous DDL studies in writing demonstrates that the following factors may affect the learning outcomes of DDL activities.

The first important factor is task type since not all writing problems can be solved equally well by DDL approach. In previous studies, DDL is adopted to learn linking adverbials (Boulton 2009; Cotos 2014), to aid learners in generating ideas and writing creatively (Kennedy and Miceli 2010), to distinguish synonym adjectives and solve learners’ problems of overusing general adjectives (Yeh et al. 2007), to learn collocation knowledge and thus help learners produce more accurate and complex language patterns (Thomas 2015). However, it seems that error-correction is the most appropriate task for DDL because a great many studies (e.g. Gaskell and Cobb 2004; Chambers and O’Sullivan 2004; O’Sullivan and Chambers 2006; Tono et al. 2014; Reynolds 2015a) focused on error-correction or self-editing in writing. For instance, Gaskell and Cobb (2004) explored the immediate effects of adopting DDL in correcting L2 learners’ sentence-level grammatical errors in writing. The results revealed that concordance evidence for lower-level learners’ grammar development was not as effective as for lexical development. Nevertheless, the results still proved the positive effects of corpus use since more than 80 % corrections were made accurately when the online concordance links were offered to them while less accurate corrections were made when removing the links. There are another two studies (Chambers and O’Sullivan 2004; O’Sullivan and Chambers 2006) focusing on the efficacy of DDL in correcting more various kinds of errors, which include lexico-grammatical errors, capitalization errors, spelling errors, etc. These findings confirmed the advantages of DDL in reducing the interference of the mother tongue, highlighting the awareness of lexico-grammatical patterns and improving the accuracy rate of error corrections. Nevertheless, not all types of errors can be corrected equally well with a DDL approach. Thus more recently, Tono et al. (2014) conducted a research on 93 upper-intermediate undergraduate students, aiming to evaluate the effectiveness of DDL in correcting three coded error types. The results revealed that omission and addition errors were easier to be identified and revised, while mis-formation errors were more difficult to be corrected accurately. These studies indicated that although DDL can be adopted to solve various writing problems, some types of tasks may be more appropriate for DDL thus teachers should carefully consider the appropriateness of tasks while organizing DDL activities.

The second significant factor is the methodology, that is, how DDL is implemented. There are generally two ways to adopt DDL approach, namely, direct DDL and indirect DDL (Yoon and Jo 2014). In direct DDL, learners consult corpora directly for solving language problems; whereas indirect DDL refers to learners’ use of paper-based concordance lines extracted by teachers or researchers. Direct DDL is the dominant paradigm in previous studies (Yoon and Hirvela 2004; Yoon 2008; Gaskell and Cobb 2004; Gilmore 2009; O’Sullivan and Chambers 2006; Pérez-Paredes et al. 2012; Chang 2014; Tono et al. 2014; Cotos 2014), but recently there are also a lot of studies employing indirect DDL such as Boulton (2009, 2010), Huang (2014), Smart (2014), etc. These studies have shown that both direct and indirect DDL have their own distinctive advantages and disadvantages. To compare the effectiveness of the two ways, Yoon and Jo (2014) conducted a small-scale study investigating their different effects on L2 learners’ error correction in a writing class. This study revealed that the self-correction rate was higher in indirect DDL than in the direct DDL for most learners, especially for lower-level learners; however, direct DDL activities may have more positive effects on learner autonomy especially for higher-level learners. Thus it could be found that indirect DDL is more appropriate for lower-level or novice learners, which is consistent with Gaskell and Cobb’s (2004) viewpoint that indirect DDL is a transitional step to direct DDL.

The third factor to be considered is language proficiency and training. It is usually believed that DDL may appeal more to advanced learners. Just as shown by O’Sullivan and Chambers (2006), the post-graduates showed more positive attitudes toward corpus use than the undergraduates. What’s more, Granath (2009) also stated that advanced learners usually benefit more from a DDL approach. Nonetheless, with proper training, DDL could be also effective for lower-level learners as well as advanced learners. As stated by Boulton (2009), for intermediate or lower-level learners, it is crucial to organize the training session to improve their corpus techniques for DDL to be successful. Therefore a lot of studies (e.g. Yoon and Hirvela 2004; O’Sullivan and Chambers 2006; Gilmore 2009; Chang and Sun 2009; Smart 2014) have designed the training session about consultation skills prior to learners’ independent use of corpora. For instance, Gilmore’s (2009) study found that the intermediate-level Japanese university students were able to significantly improve the naturalness of their redrafted essays with a merely 90-min training about how to use online corpora. Chang and Sun (2009) also showed students performed better in the revision tasks with the aid of scaffolding prompts or teachers’ instructions. In Yoon and Hirvela’s (2004) study, due to receiving more direct training and practice in corpus use, the intermediate students reported fewer problems in corpus search techniques than the advanced students, and they showed more positive attitudes towards corpus use. Although the study conducted by Boulton (2009) proved that lower-level learners could also benefit a lot from DDL in learning linking adverbials in writing without prior training, an overwhelming majority of studies reveal that appropriate training is helpful to maximize the advantages of DDL activities for both lower-level and higher-level students, especially for Asian students who are not accustomed to this exploratory learning approach.

The last factor that can be never ignored in the implementation of DDL is the choice of corpora or other Internet resources. The size and type of a corpus may determine the effectiveness of DDL activities. A large and general corpus, such as the Collins COBUILD corpus and British National Corpus (BNC), is often regarded as a good choice for DDL activities due to the massive authentic examples of different usages it could provide. Thus, some studies (e.g. Yoon and Hirvela 2004; Yoon 2008; Gilmore 2009) selected general corpora as reference resources in DDL activities. Some others (e.g. Kennedy and Miceli 2010; Lee and Swales 2006; Chang 2014) preferred to use specialized corpora in academic writing, because this type of corpora can usually provide more effective information about the genres and disciplines in which L2 learners have to write. Charles (2014) even had learners compile their own corpus, which is proved to be useful in helping learners “get the corpus habit” in writing. In Reynolds’ (2015b) study, 25 Taiwanese medical students were encouraged to exploit a web-based English/Chinese bilingual parallel corpus collocational concordancer for self-editing their academic writing. In addition to the above-mentioned corpora, web as a corpus and concordancer has also attracted a lot of attention and the search engine is suggested as a super corpus in some studies (Resnik and Smith 2003; Resnik and Elkiss 2005; Sha 2010; Conroy 2010; Brezina 2012; Yoon 2016). These studies revealed the distinctive merits and demerits of different corpora, thus it’s rather difficult to conclude which type of corpora is better. Although some studies (Chang 2014; Sha 2010) compared the usefulness of different types of corpora, this type of empirical studies are relatively few. It’s necessary to find out the appropriate corpora as reference resource for learners. In reality, with the increasing availability of Internet access, the readily available general purpose corpora like BNCweb and the search engine familiar to students are good choices for ordinary teachers and students. BNCweb relies on the Corpus Query Processor (CQP) of the IMS Open Corpus Workbench, providing user-friendly and powerful interface to students (Hoffmann et al. 2008). It is freely accessible to everyone with an Internet connection and enables students who are poor in corpus techniques to exploit corpora just like browsing web pages. The advantages of search-engine-based corpora such as the Linguist’s Search Engine and Google are also obvious due to their usability, search speed and the number of solutions. Learners with different cultural background need to be provided with different reference tools, thus empirical studies are needed to find out the appropriate reference resource for specific learners.

From the above analysis of previous DDL studies, it becomes clear that task type, methodology, language proficiency and training as well as the choice of corpora are important variables to be considered while designing DDL activities. It is also obvious that the carefully designed DDL activities are effective in helping learners solve some writing problems. Nevertheless, there are still some limitations of these studies. Firstly, most of the studies just focus on the immediate effects of DDL in classroom settings or computer labs such as improving the accuracy of error-correction. However, as remarked by Boulton (2011), the biggest advantage of DDL lies in medium-term or longer-term benefits. Secondly, there is a lack of empirical studies comparing the effectiveness of different types of corpora in DDL activities. To complement existing research and to aid teachers in presenting future guidance to students, the present study conducts an empirical study on EFL learners in mainland China, aiming to achieve the following purposes. Firstly, it intends to explore whether the independent DDL activities outside the classroom over a whole term can facilitate EFL learners’ writing development in terms of fluency, accuracy and complexity. Secondly, to compare which type of corpora is more useful for Chinese EFL learners in DDL activities. Thirdly, to investigate EFL learners’ perceptions of BNCweb-assisted DDL activities.

## Methods

### Research questions

By taking into account all the variables that may influence the effectiveness of DDL activities, the research is carefully designed and it is guided by the following questions.

1. Are there significant differences in the writing fluency, accuracy and complexity before and after the adoption of BNCweb-assisted DDL activities?
2. Which type of corpora, BNCweb or the search engine Baidu, is more useful for Chinese EFL learners in DDL activities to facilitate the writing development in terms of fluency, accuracy and complexity?
3. What are Chinese EFL learners’ perceptions of adopting BNCweb-assisted DDL approach in revising compositions?

### Participants

The participants in this study were Chinese freshmen of non-English majors from a science and engineering university in western China. They had studied English at school for at least 6 years before entering higher education and this term they were enrolled in a compulsory College English course. Although 51 students participated in this experiment at the beginning, 3 students were absent for several times, failing to complete and hand in some of the required writing tasks on time. Thus, they were excluded from the study. Finally, in total, 48 students’ data were collected following the experimental session. These students were divided into two groups. The 26 participants in the experimental group, including 20 males and 6 females, were from one College English class; whereas the other 22 participants in the control group, including 18 males and 4 females, were from another parallel English class. These participants were labeled as intermediate-level learners, according to their performance in the College entrance examination, with English scores ranging from 106 to 128 out of a maximum 150. The English placement test before College English course showed that students in the experimental and control group were equivalent in the overall English proficiency. These participants were all told that if they strictly follow the instruction and finish the designated writing and redrafting assignments on time, they could get additional scores for this course.

### Instrumentation

#### A writing test

The topic of the writing in the pre-test and post-test, my view on cell-phones, is given to students. They were all familiar with cell phones, thus it is not difficult for them to pool ideas and to compose their own work under time constraints. Both the pre-test and post-test should be finished within 30 min and they were encouraged to write as much as possible. No references were allowed for basic references.

#### BNCweb (CQP-edition)

**Fig. 1:**
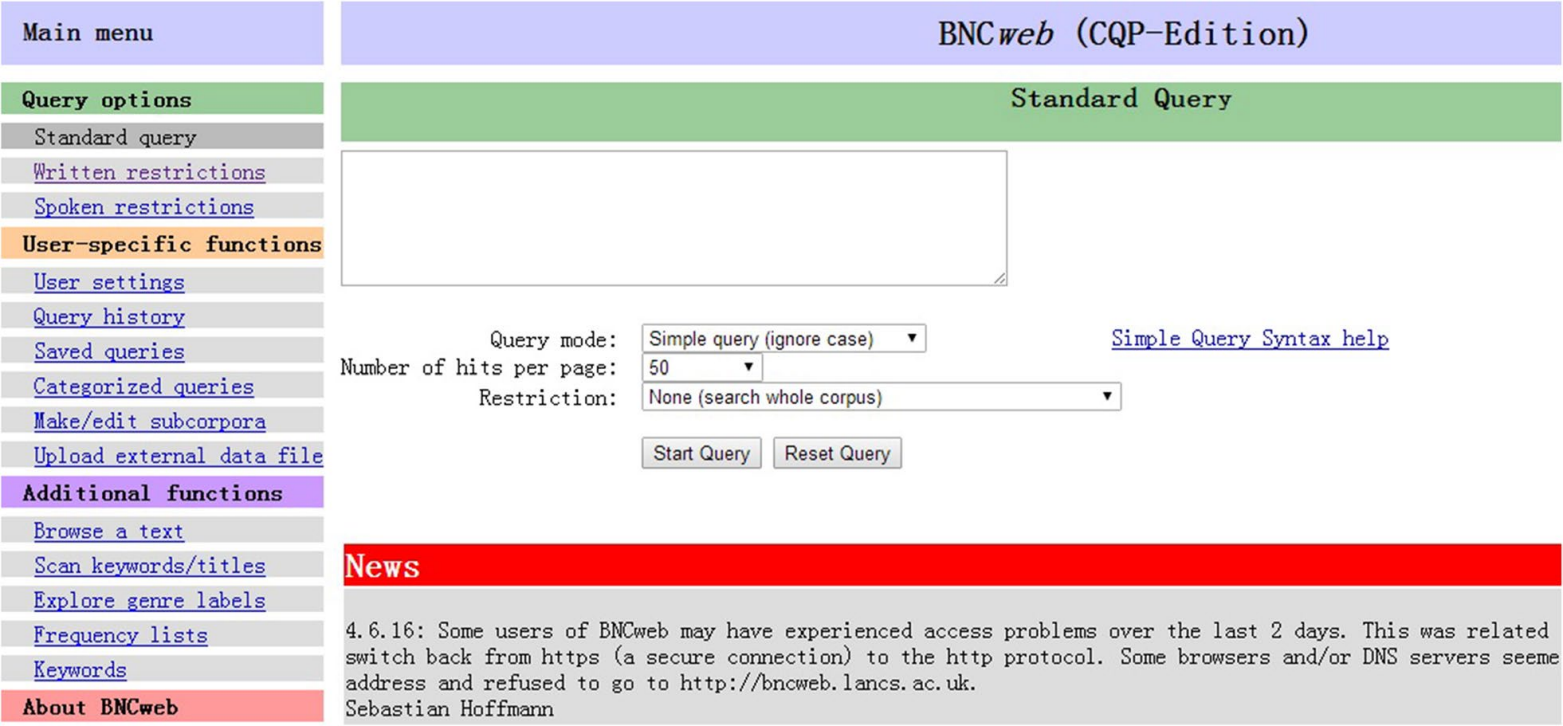
The screenshot of BNCweb (CQP-edition) interface

CQP web, as the fourth generation corpus analysis tool, combines ease of use, power and flexibility to a very high degree by making a complex query system accessible to users without special training (Hardie 2012). A particular advantage of CQP is the ability to perform very general searches on large corpora and efficiently deal with millions of hits they may return. In addition, any query can be saved within CQPweb, or downloaded to the user’s computer as a plain-text table (Hardie 2012). The original version of BNCweb used a SARA back-end, but now it has evolved significantly to use the CQP back-end, which dramatically increases the speed of the system. The new version of BNCweb (http://bncweb.lancs.ac.uk/) combines the efficiency and flexibility of CQP queries with the user-friendliness of BNCweb (Hoffmann and Evert 2006). The screenshot of BNCweb interface can be seen in Fig. 1.

The CQP edition of BNCweb was applied in the present study to assist the students in the experimental group to redraft essays for the following advantages. Firstly, it’s fast due to the fact that it just takes seconds to obtain a collocation analysis of more than 20 thousand instances in the BNC. Secondly, it’s simple to use and flexible. Students can not only perform simple queries just by typing in a single word or a sequence of lexical items, but also conduct more complex searches by using CQP syntax (Hoffmann et al. 2008). This means students can easily get the high-frequent adjective or adverb collocates of target words, which to some extent can solve students’ problems of under-using adjectives or adverbs and improve the lexical richness of their writing. Furthermore, the advanced CQP query syntax can assist students to get some special sentence structure, which provides guidance for redrafting sentences. Thirdly, it provides a whole range of features for corpus analysis, such as concordance display, sort, collocations, distribution analysis, etc. (Hoffmann and Evert 2006). Fourthly, it is freely accessible to everyone with an Internet connection. Fifthly, it can be simultaneously used by thousands of learners. In this study, BNC was mainly used for two types of revision tasks, including error correction and rewritten work, to make their essays more accurate, fluent and complex.

#### Search engine Baidu

**Fig. 2:**
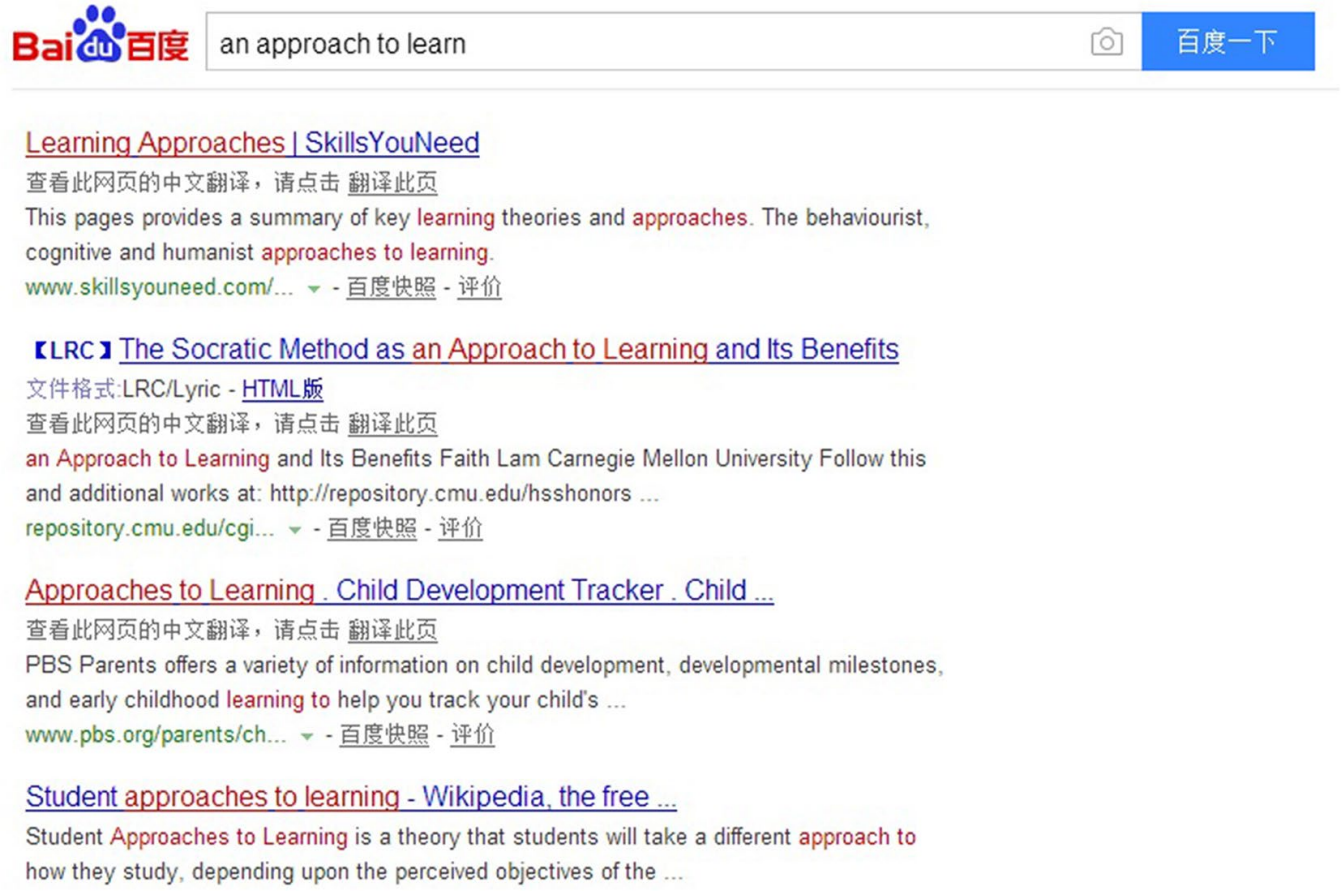
The screenshot of the results of simple query for “an approach to learn”

Strictly speaking, the search engine Baidu is not a conventional corpus. Nevertheless, according to Sha’s (2010) statement about the requirements for appropriate corpora in DDL activities, it could be used as reference resources. While using it, the Internet serves as the body of machine-readable text and the search engine serves as the concordancer. Baidu is by far the most-commonly used search engine in China. In the survey prior to the experiment, 90 % of students indicated that they spontaneously turn to Baidu in search of linguistic help when they encountered problems in writing. Thus Baidu (http://www.baidu.com) is selected as the reference resource for the students in the control group. Although Baidu is a Chinese language search platform, students can get access to millions of web pages which can provide many authentic English usages and expressions by simply entering words, phrases or expressions. For instance, when the wrong English phrase “an approach to learn” is typed in, a lot of web pages containing various forms of this phrase (see Fig. 2) may appear including the correct usage an approach to learning English which may provide guidance for revising or redrafting sentences.

In most of the cases, students can get bilingual information about language use. In addition, by typing in the topic of the writing, students could get a lot of excellent sentences or even articles which could help them polish their compositions. Furthermore, through Baidu, students can get access to a lot of online dictionaries, such as Youdao dictionary (http://dict.youdao.com). In this study participants in the control group were required to use Baidu in the redrafting stage to correct errors and rewrite phrases or sentences so as to improve the fluency, accuracy and complexity of their essays in the routine writing assignments.

#### Juku Grading system

The Juku Grading system is a software with which learners can be provided with individualized and intelligent assessment in China. Juku Grading Net (http://www.pigai.org/) has been in the dock for several years in universities and its scores whose accuracy rate is over 90 % enjoy a high consistency with that of human raters.

After collecting all the essays, the researcher typed them into the computer and then submitted them to Juku Grading system. This Grading system can help calculate the total words of each essay and identify the errors. Errors including collocation errors, verb errors, noun errors, word choice errors, run-on sentences, etc. were identified by Juku and then counted by the researcher. In addition, Juku could calculate TTR (type–token ratio) automatically which can provide data for the measurement of vocabulary complexity.

### Data-collection procedures

In the present research, a pre-test/post-test experimental and control group design was adopted; that is to say, the participants in the two groups were all asked to take part in a pre-writing test in class, then the treatment, and then a post-writing test which is the same as the pre-test. The experiment was conducted with two groups of freshmen from two parallel English classes of non-English majors. In the College English course, the teacher met them twice a week. The whole experiment was divided into three stages: the pre-treatment stage, the during-treatment stage, and the post-treatment stage.

In the pre-treatment stage, both the experimental group and the control group took a pre-writing test. And then participants in the experimental group received training on how to consult BNCweb as reference resources to revise sentences in writing, while the control group received instruction on how to use the search engine Baidu to redraft or self-edit sentences. The training for the experimental group consists of three parts. The first part is the introduction about what a corpus is and what functions of BNCweb can be used to help students revise essays. In the second part, students were guided to utilize BNCweb for correcting errors or polishing sentences in writing, aiming to help students master the consultation skills including some simple Part-of-Speech (POS) tags. In the third part, students were given some sentences from the written part of Spoken and Written English Corpus of Chinese Learners (SWECCL) to revise or polish. Since many participants were not able to identify some errors or inappropriate expressions, the erroneous part that should be revised or polished were underlined. In this training session, indirect DDL was mainly adopted in the classroom for helping learners analyze and induce rules from concordance lines and direct DDL was also used by learners outside the classroom. For the control group, the training was mainly the guidance about how to obtain and distinguish reliable and accurate information from the Internet. Because learners just need to type in the key words, phrases or sentences in Baidu, there were no typical consultation skills to learn. The revising materials for the two groups to practice in the training session were the same and the only difference was the consultation tools. After the training, the participants in both groups were able to complete the following two types of tasks with their designated reference tools. The first type of the task was to correct errors so as to improve the accuracy of the sentences. The second type of the task was to polish the sentences in writing to make the original sentences longer or improve the complexities of the sentences, which is mainly achieved by adding some components such as adjectives, adverbials or even clauses, or by replacing the original simple words with more accurate and complex expressions.

In the during-treatment stage, participants in both groups were required to write compositions and revise the underlined parts marked by the researcher through utilizing different reference resources. The experimental group consulted BNC for revision, whereas the control group utilized Baidu to improve their writing. In total, all the participants should finish five compositions and the relevant revision work. Every composition was finished according to the following procedure. Firstly, the participants finished the writing assignments and then handed them in. Secondly, their compositions were marked by underlining the parts which should be rewritten or corrected and then the participants in the two groups revised the composition out of class with the assistance of their own reference resources respectively. To ensure that they really consulted BNCweb or Baidu, they were asked to provide the url links or other stored information while turning in the revised version. Thirdly, their revised compositions were collected and a comment was given on the revision work to each participant by confirming the right revision and correcting the inappropriate revision. For those unable to exploit BNCweb or Baidu well for revision work, the individual guidance was offered to them after class in order to ensure that all of them could fully utilize the consultation tools.

In the post-treatment stage, all the participants were requested to take part in the post-writing test in class with the same amount of time and the same topic as the pre-writing test. To obtain information about the effects of corpus use on EFL learners’ writing development from learners’ perspective, an individual interview was performed on ten participants who were randomly chosen from the experimental group. To make them express their ideas better, the interview was conducted in Chinese.

### Data analysis procedures

In this study, quantitative and qualitative research methods were employed to explore the research questions. To answer the first two research questions, the compositions collected in pre-test and post-test were analyzed for fluency, accuracy and complexity. This was achieved by calculating the total words of each composition and then dividing each composition into T units and clauses, and identifying dependent clauses (Wigglesworth and Storch 2009).

Fluency was measured by the average number of words per composition (Storch 2005), which was done by Juku automatically providing the total words of each essay.

Accuracy was measured in terms of the proportion of error-free T-units of all T-units (EFT/T) and errors per 100 words (total number of errors/total number of words × 100). A T-unit was defined by Larsen-Freeman (2006) as “a minimal terminal unit or independent clause with whatever dependent clauses, phrases and words are attached to or embedded within it”. Independent clauses and dependent clauses were defined in the same way as Wigglesworth and Storch’s (2009) study. Errors in this study were mainly identified by Juku, including collocation errors, verb errors, article errors, preposition errors, word choice errors, subject-verb disagreement, sentence structure errors, etc. Word choice errors were included only when they impede meaning. Punctuation errors and spelling errors were excluded since these types of errors were not regarded as severe problems that may impede understanding.

Complexity was measured by vocabulary complexity and grammatical complexity. Vocabulary complexity was based on a sophisticated type-token ratio (Larsen-Freeman 2006), which is calculated by Juku Grading system automatically. Grammatical complexity was measured in terms of the average number of clauses per t-unit. These measures have been regarded as reliable and effective in evaluating second language development in writing (Larsen-Freeman 2006).

After getting the specific data about these essays, an independent-samples t-test was conducted with the aid of SPSS 17.0 to examine whether there were significant differences between the experimental group and the control group in terms of fluency, accuracy and complexity. For the purpose of evaluating the effects of using BNC CQP web in the revising stage, the results from the pre-test and post-test measurements in the experimental group were calculated and a paired samples T-test was conducted.

Finally, to answer the third research question, a semi-structured interview technique was utilized in the experimental group and tape recording was adopted during the process of interview. After the interview, the interviewees’ answers were transcribed so as to explore students’ perceptions regarding their use of BNC CQP web while revising essays. Then all the data were carefully studied to figure out the similarities and differences.

## Results

The compositions collected in this research are analyzed quantitatively based on the measures described above and the interview data were analyzed qualitatively. The results are presented in the following sections.

### Comparison of compositions for the pre-test and post-test

The following three tables present the paired samples T-test results about the comparison of compositions produced by the experimental group in the pre-test and post-test.

As illustrated in Table 1, the significant difference exists between the pre-test and post-test in terms of writing fluency in the experimental group, which can be seen from the p value at 0.000. And M value increases from 135.08 to 190.19, showing that participants in experimental group produced obviously longer compositions in the post-test as compared with the pre-test. It is possible that participants have increased their vocabulary size in the process of revising essays with the aid of BNC web, because it’s commonly believed that the large vocabulary size can help increase the length of the essays.

**Table 1:** Measure of fluency for essays in the pre-test and the post-test

Group	Test	N	M	SD	95 % confidence interval of the difference		t	Sig.(2-tailed)
					Lower	Upper		
Ex.	Pre-test	26	135.08	21.735	−73.4563	−36.7744	−6.189	0.000*
	Post-test	26	190.19	40.634				

*M* mean number of words per composition

* p < 0.05

From Table 2, it can be found that experimental group performed significantly better in the post-test in terms of writing accuracy compared with the pre-test. On the one hand, the proportion of error-free T-units to T-units increased significantly from 0.4481 to 0.6200 (p = 0.000), showing that participants wrote more accurate sentences after the adoption of DDL in the revision stage; on the other hand, errors per hundred words decreased significantly from 8.1758 to 4.9296 (p = 0.000), indicating that the learners made much fewer errors in the post-test.

**Table 2:** Measures of accuracy for essays in the pre-test and the post-test

Group	Accuracy	Test	N	M	SD	95 % confidence interval of the difference		t	Sig.(2-tailed)
						Lower	Upper		
Ex.	EFT/T	Pre-test	26	0.4481	0.1715	−0.1950	−0.1612	−21.692	0.000*
		Post-test	26	0.6200	0.1580				
	E/W × 100	Pre-test	26	8.1758	2.6419	1.9078	4.5845	4.996	0.000*
		Post-test	26	4.9296	3.0076				

*EFT/T* error free T-units/T-units, *E/W* × *100* errors per 100 words

* p < 0.05

It can be revealed from Table 3 that participants’ performances in the pre-writing and post-writing test are similar in both of the two measures of complexity. The results demonstrate that the use of BNC web in revision stage may have no obvious positive effects on EFL learners’ writing development in terms of complexity, at least on the two measures described above.

**Table 3:** Measures of complexity for essays in the pre-test and the post-test

Group	Complexity	Test	N	M	SD	95 % confidence interval of the difference		t	Sig.(2-tailed)
						Lower	Upper		
Ex.	TTR	Pre-test	26	5.5104	0.8483	−0.6384	0.4630	−0.328	0.746
		Post-test	26	5.5981	0.8662				
	C/T	Pre-test	26	1.6258	0.3561	−0.2433	0.9328	−0.918	0.367
		Post-test	26	1.7008	0.2446				

*TTR* type–token ratio (word types per square root of two times the words), *C/T* clauses/T-units

### Comparison of compositions by experimental group and control group

The independent samples T-test results about the comparison of compositions completed by experimental group and the control group can be seen in Tables 1, 2 and 3.

As shown in Table 4, the experimental group produced much longer essays than the control group in the post-test writing, with a significant difference at the 0.05 probability level (p = 0.020). However, no identifiable difference exists between the two groups in the pre-test writing (p = 0.625) in terms of the mean number of words per composition. The results reveal that BNCweb as reference resource in the revising stage is better than Baidu search engine in helping EFL learners improve their writing fluency. Fluency is defined by Wolfe-Quintero et al. (1998) as “the amount a writer produces in a specific time period”. Being given the equal amount of time, participants in the experimental group wrote 190.19 words on average while those in the control group just produced 164.95 words on average. This demonstrates that participants in the experimental group wrote more fluently than those in the control group.

**Table 4:** Measure of fluency in compositions by experimental group and control group

Test	Group	N	M (no. of words)	SD	Mean diff.	t	Sig.(2-tailed)
Pre-test	Ex.	26	135.08	21.735	−3.378	−0.492	0.625
	Control	22	138.45	25.836			
Post-test	Ex.	26	190.19	40.634	25.238	2.406	0.020*
	Control	22	164.95	30.115			

*M* mean number of words per composition

* p < 0.05

In Table 5, the measures for accuracy show that there is no significant difference for either EFT/T (p = 0.536) or errors per words (p = 0.801) in the pre-writing test, whereas there are statistically significant differences in both EFT/T (p = 0.004) and errors per words (p = 0.006) in the post-writing test. The results demonstrate that participants in the experimental group produced much more accurate sentences and made significantly fewer errors, compared with those in the control group, after the treatment of using different web resources in the redrafting stage. Therefore, BNC web, as reference resource in revision activities, has more effective effects on EFL learners’ writing development in terms of accuracy, at least on the measures adopted in this study.

**Table 5:** Measures of accuracy in compositions by experimental group and control group

Test	Accuracy	Group	N	M	SD	Mean diff.	t	Sig.(2-tailed)
Pre-test	EFT/T	Ex.	26	0.4481	0.1715	0.2899	0.624	0.536
		Control	22	0.4191	0.1458			
	E/W × 100	Ex.	26	8.1758	2.6419	−0.2047	−0.253	0.801
		Control	22	8.3805	2.9525			
Post-test	EFT/T	Ex.	26	0.6200	0.1579	0.1386	2.991	0.004*
		Control	22	0.4814	0.1625			
	E/W × 100	Ex.	26	4.9296	3.0076	−2.5536	−2.910	0.006*
		Control	22	7.4832	3.0546			

*EFT/T* error free T-units/T-units, E/W × 100 = errors/hundred words (total number of errors/total number of words × 100)

* p < 0.05

Table 6 indicates that there are no identifiable differences between the essays produced by the two groups in terms of writing complexity in the pre-writing test as well as the post-writing test. Complexity, in this study, is measured based on the vocabulary complexity (a sophisticated TTR) and grammatical complexity (Clauses/T-units). The independent–samples t-test reveals no significant difference in both of the two measures, indicating that BNCweb has no obvious positive advantages over Baidu in developing EFL learners’ writing complexity.

**Table 6:** Measures of complexity in compositions by experimental group and control group

Test	Complexity	Group	N	M	Std.	Mean diff.	t	Sig.(2-tailed)
Pre-test	Type-token ratio	Ex.	26	5.5104	0.8483	−0.1010	−0.442	0.661
		Control	22	5.6114	0.7116			
	Clauses/T-units	Ex.	26	1.6258	0.3561	0.0458	0.515	0.609
		Control	22	1.5800	0.2352			
Post-test	Type-token ratio	Ex.	26	5.5981	0.8662	−0.1097	−0.431	0.668
		Control	22	5.7077	0.8923			
	Clauses/T-units	Ex.	26	1.7008	0.2446	−0.0561	−0.556	0.581
		Control	22	1.7568	0.4406			

*TTR* type–token ratio (word types per square root of two times the words)

### Students’ perceptions of BNCweb-assisted DDL approach in revising compositions

Ten students randomly selected from the experimental group were interviewed in Chinese about their perceptions of DDL activities in revising stage. The qualitative interview results indicate that the interviewees generally show positive attitudes toward the use of BNC web in revision stage of writing. They emphasized that the use of a corpus in the revising stage can help them improve their writing accuracy because the time-consuming DDL activities make them have a better impression of their errors and the correct language patterns. In DDL, it is usually the learners who explore and find out appropriate expressions or collocates relevant to his or her writing. This discovery-oriented process may facilitate their acquisition of the correct language use. The interviewees here show the same opinion with the participants in Chambers’ (2005) study, who claimed that hunting for grammatical patterns in concordance lines better facilitates memorization of problematic aspects of the target language than being “spoon-fed” the language rules. In addition, most of them mentioned that the frequently-occurring collocations presented in Key-Word-in-Context (KWIC) format help them increase the awareness of collocation and thus enables them to use these collocations better in the writing tasks. Due to the interference of the mother tongue, they often associate a verb with a noun wrongly or match a noun arbitrarily with an adjective without considering whether it is appropriate. Now after the corpus consultation activities, they realized it’s better to memorize the collocations or phrases in context rather than the single word. Besides these benefits of DDL activities, some of them still mentioned the following advantages such as discovering some new usages of the known words accidentally, learning other new words incidentally, making them become more autonomous, enlarging their vocabulary.

However, not all types of learners like this learning approach (Gilmore 2009), there are still some learners tired of using corpora for revision tasks by saying that “I like Baidu better since I can usually get a direct answer about how to correct errors by simply typing in an expression or a sentence.” Many Chinese EFL learners have already been accustomed to being directly told how to correct errors in writing in their prior school career, so the new exploratory approach may not appeal to them. Nevertheless, a majority of the interviewees responded that “we are more willing to use corpora in many cases and will continue to use it in the future writing”. When being asked about the problems they encountered while using corpora for revision tasks, almost all of them mentioned that they often feel frustrated when failing to get what they want after doing several queries in the corpora. They emphasized that “lack of concordance technique” and “lack of ability to induce language rules” brought trouble to them. Furthermore, they still expressed that they can hardly get the desirable results when trying to find out an English equivalent for an expression in Chinese. Just as stated by Sha (2010), DDL approach is not effective in helping learners paraphrase exactly what they try to express in a second language. This is really a limitation of merely using corpora in DDL, and maybe it can be solved by integrating other reference resources into corpora. Although time-consuming is another problem mentioned by them, most of them think it’s OK because they consulted corpora out of class which makes them make better use of their free time, or they may waste it in doing some other meaningless things.

To sum up, most of the interviewees believed that the benefits outweighed the problems of DDL approach and the use of a corpus for revising essays could help them improve their overall writing quality.

## Discussion

The quantitative results of the present study reveal that DDL activities in the redrafting stage have significantly positive effects on EFL’ learners’ writing development in terms of fluency and accuracy. In addition, the online corpus BNCweb is obviously better than the search engine Baidu in developing learners’ writing fluency and accuracy. Nevertheless, no obvious advantages of BNC web have been found in terms of writing complexity, compared with Baidu.

Fluency is one of the aspects that can show a learner’ writing competence. To explore the reasons for the significant difference in fluency between the two groups, a careful observation of the essays produced in the post-test is done. It’s found that participants in the experimental group tend to use collocations more frequently compared with those in the control group. For instance, they wrote “have both positive and negative impacts on us” instead of “impact us a lot”, “make up one’s mind to do” instead of “decide to do”, etc. An obvious advantage of DDL activities is to promote learners’ awareness of collocation. In the process of using BNCweb, learners can gain numerous concordance lines which can present various usages of target words or phrases. As some interviewees expressed, the recurrent collocations or phrases make them naturally notice and thus remember the language forms. This provides support for the studies conducted by Chambers and O’Sullivan (2004) and O’Sullivan and Chambers (2006), which claimed that the use of corpus in writing can help learners notice and learn lexico-grammatical patterns. And this may be also a reason to explain why students in the experimental group produced significantly longer essays in the same amount of time. In addition, participants in the experimental group used more adjectives or adverbs, such as “extremely useful”, “strongly suggest”, “benefit enormously”. In the during-treatment stage, learners were required to add some words or components to the original sentences in writing. Participants in the experimental group usually achieved this by searching the high-frequent adjective or adverb collocates of a specific word in BNCweb. Some high-frequent adjectives or adverbs were thus kept in students’ mind, which may reoccur to them in the post-test. Furthermore, learners’ continual interaction with corpus data also aid students to incidentally acquire some new words. In this process, students’ vocabulary size has been increased, which to some extent helps them produce essays more fluently.

Accuracy is also a main factor that may affect instructors’ assessment of L2 learners’ writing (Yoon 2008). Students in the experimental group made obvious improvement and behaved much better than learners in the control group in writing accuracy. This result is consistent with Luo and Liao’s (2015) study that the persistent use of corpora in revision tasks can help learners reduce errors in writing. As stated by Corder (1981), making learners try to discover the correct language patterns could often be more instructive and helpful to them. The participants who used BNC web usually spend more time in discovering the right expressions and correcting errors which may make them have a better memory of the errors and the correct usages retrieved from the corpora. While writing, they may naturally recall the right usages and avoid the similar errors. But participants in the control group sometimes just type in their erroneous expressions in Baidu, and then they may get a response directly about how to correct them without deep consideration. As indicated by Reynolds and Anderson (2015), passive students seldom review error corrections to a degree that is necessary to internalize natural patterns of the written language. Thus they may commit the same errors in the next writing assignment especially the errors due to the interference of mother tongue. Furthermore, as indicated by Geluso (2013), native speakers of a language are keenly aware of formulaic language largely due to frequency effects, while DDL can provide useful insights into frequent patterns of authentic language to EFL students. These highly frequent language patterns can undoubtedly help these participants produce more accurate and native-like expressions.

Complexity is another significant aspect that can reveal a learner’s writing competence, thus it is also measured in case that some learners are reluctant to try to produce more complex words or sentences for fear of making errors. The results reveal that the group adopting a DDL approach didn’t make obvious improvement in both of the vocabulary complexity and grammatical complexity. Furthermore, according to some interviewees, Baidu seems to help them better in improving writing complexity since students can usually get a lot of relevant complex sentences or even excellent essays just by simply typing in the topic of the essay in Baidu in the process of revising essays. However, they often have to do complex query in BNCweb for getting the desirable sentences. In reality, many students just use the simple query function by simply typing in some phrases, but rarely use POS tags offered to them while consulting corpora. This is consistent with Pérez-Paredes et al.’s (2012) discovery that learners just used BNC as if they were using Google without using POS tags or regular expressions. Therefore, the lack of corpus consultation skills is an important factor that may influence learners’ improvement in complexity. Just as stated by Sabti and Chaichan’s (2014) that computer skill is the biggest barrier that impedes the use of computer technologies in language learning. Thus more training or instruction about consultation skills should be provided to students. With good consultation skills, students will exploit corpora better thus they may easily get the complex sentence structure that they want from BNCweb while revising sentences. Then in the writing test they may be able to produce complex sentences. In addition, it usually takes a rather long time for one to significantly improve one’s productive use of vocabulary and grammatical knowledge. Thus it is suggested that teachers continue the DDL activities in writing for a longer time period, and then evaluate their writing complexity again. Moreover, EFL learners should be encouraged to take risks to produce more complex words or structures and not to be afraid of making errors in regular writing assignments. In the process of dealing with complex expressions or structures, their language proficiency will be improved, which may result in their better performance in complexity in writing test.

## Conclusion

The present study explored the effects of DDL activities on Chinese EFL learners’ writing development in terms of fluency, accuracy and complexity. The results confirmed the effectiveness of adopting DDL in redrafting essays in developing learners’ writing fluency and accuracy, whereas no statistical evidence was found about the usefulness of DDL in improving learners’ writing complexity. In addition, the results revealed the obvious advantages of BNCweb over the search engine Baidu in improving learners’ writing fluency and accuracy. Nevertheless this doesn’t mean that DDL activities are completely useless in helping learners produce complex essays. It usually takes a rather long time to improve one’s writing quality in terms of complexity, but this study just lasts for an academic term which is not long enough. Thus, a more longitudinal study should be conducted about the influence of DDL on EFL learners’ writing competence especially in complexity. Furthermore, the web-based EFL writing platform such as IWILL 2.0 (Reynolds 2013) can be used to provide instant electronic feedback instead of teachers’ marking before requiring students to redraft essays in the future study, which would be more convenient for teachers and students.

Students being interviewed generally expressed positive attitudes toward the application of DDL in revising compositions but there were still some students claiming that DDL activities sometimes decrease their motivation. Teachers are suggested to design appropriate DDL tasks based on individual learners’ needs and analytical abilities; in addition, more training and guidance should be offered to help them overcome the technical and psychological barrier. In addition, to counter the potential barriers of using corpora in writing, the combined use of different types of reference resources in DDL activities should be considered. As stated by Yoon (2016), using concordancing tools along with other complementary reference resources within a single interface may help advanced L2 writers more effectively in writing. Thus further study can be conducted to explore the effectiveness of the combined use of different reference resources in writing.
